# Determinants of intermittent preventive treatment of malaria among women attending antenatal clinics in primary health care centers in Ogbomoso, Oyo State, Nigeria

**DOI:** 10.11604/pamj.2019.33.101.14800

**Published:** 2019-06-11

**Authors:** Adefisoye Oluwaseun Adewole, Olufunmilayo Fawole, IkeOluwapo Ajayi, Bidemi Yusuf, Abisola Oladimeji, Endie Waziri, Patrick Nguku, Olufemi Ajumobi

**Affiliations:** 1 Nigeria Field Epidemiology and Laboratory Training Program (NFELTP), Abuja, Nigeria; 2 Department of Community Medicine Ladoke Akintola University of Technology Teaching Hospital (LAUTECH) Ogbomoso, Oyo State, Nigeria; 3 Department of Epidemiology and Medical Statistics, Faculty of Public Health, University of Ibadan, Nigeria; 4 National Malaria Elimination Program, Federal Ministry of Health, Abuja, Nigeria

**Keywords:** Malaria, intermittent preventive treatment, pregnancy, primary healthcare centres, knowledge

## Abstract

**Introduction:**

Despite the effectiveness of intermittent preventive treatment in pregnancy using sulphadoxine-pyrimethamine (IPTp-SP), the uptake and coverage in southwest Nigeria are low. We assessed the factors influencing utilisation of IPTp-SP.

**Methods:**

A multistage sampling technique was used to select 400 pregnant women from six primary healthcare centers in Oyo State. Data on socio-demographic characteristics, knowledge, attitude towards IPTp-SP and its utilisation were obtained using a semi-structured questionnaire. Data were analyzed using SPSS software. Focus group discussions (FGD) and key informant interviews (KII) were held for pregnant women and healthcare workers and analysed thematically.

**Results:**

Mean age of respondents was 27.2 (SD ± 5.5) years. Mean gestational age was 29.5 weeks (SD ± 5.4). Overall, 320 (80.0%) took SP, of which 152 (47.5%) took 2 doses and 112 (35.0%) took under directly observed therapy (DOT). We found that early booking for ANC, more than two visits to ANC (adjusted odds ratio (aOR) = 5.6; 95% CI: 1.2 - 26.6), good knowledge on IPTp (aOR = 9.3; 95% CI: 5.4 - 16.0), positive attitude towards IPTp (aOR = 2.1; 95% CI: 1.5 - 2.9) and being employed (aOR = 1.4; 95% CI: 1.1 - 1.7) were factors associated with IPTp-SP utilisation. The FGD and KII revealed that IPTp-SP drugs were mostly taken at home due to stock out.

**Conclusion:**

Late ANC booking with stock out of IPTp-SP drugs was responsible for its low utilisation. There is need to encourage pregnant women to book early for ANC. Adherence to the practice of DOT scheme is recommended to improve IPTp-SP utilisation.

## Introduction

Malaria is a major public health problem with the greatest impact of its burden being in sub-Saharan Africa which accounts for 90% of the global deaths [1]. Most of the burden of disease occurs among populations at highest risk namely; pregnant women and infants [2, 3]. Malaria in pregnancy causes up to 10,000 maternal deaths each year and contributes to high rates of maternal morbidity especially in first-time mothers [4, 5]. Also, 75,000 to 200,000 infant deaths annually are attributable to malaria in pregnancy [3, 6]. Malaria in pregnancy increases the risk of miscarriage, stillbirth and low birth weight [2, 3]. The World Health Organisation (WHO) and national guideline recommendations on malaria infection during pregnancy include use of intermittent preventive treatment with sulphadoxine-pyrimethamine (IPTp-SP) at each routine antenatal clinic (ANC) visit at least one month apart after the first trimester till delivery [6]. It should be administered under supervision during ANC visits. In Nigeria, ANC clinics are considered an important entry point to target pregnant women for care as 60-70% of women attend ANC clinic at least once during any pregnancy [7, 8]. The 2013 Nigeria Demographic and Health Survey (NDHS) showed that 15% of pregnant women received two doses of SP with at least one dose administered during an ANC visit [9]. The 2015 Malaria Indicator Survey (MIS) however reported an increase from those that received at least one dose (47% of women) and three or more doses (19% of women) [10]. In the south-west zone of Nigeria, there has been an increase of pregnant women that received at least a dose, two or more and three or more doses of IPTp-SP from 23%, 10.5% and 4.3% to 64%, 54% and 20% respectively [9, 10]. In Oyo State, 11% of pregnant women received any SP during an ANC visit while 3% took two or more doses of SP and 1.9% three doses or more of SP [9]. To address this inadequacy, identification of determinants of IPTp-SP utilisation is critical to the institution of control efforts. We carried out a study to assess factors influencing the utilisation of intermittent preventive treatment in pregnancy in Ogbomoso, Oyo State, Nigeria.

## Methods

**Study area:** Ogbomoso town is situated about 600m above sea level with a mean annual temperature of about 26.2°C. The vegetation is a derived savannah region. Ogbomoso has the tropical wet and dry climate as it falls in the transition zone of guinea-savannah. The region experiences a fairly high uniform temperature, moderate to heavy seasonal rainfall. The relative humidity is within the range 75-95% [11, 12]. Stable malaria transmission occurs in the south-west region with a prevalence of 16.6% [10]. The study sites were Ogbomoso North and South Local Government Areas (LGAs). Ogbomoso north has an estimated total population of 243,400 persons with women of child bearing age constituting 53,548 [13]. Ogbomoso south has an estimated total population of 122,863 persons with women of child bearing age constituting 27,029. There are 12 primary health facilities in Ogbomoso North LGA. Ogbomoso South has 13 primary health facilities. A total of 10 Primary Health Care Centers (PHCs) (5 PHCs each) in both study LGAs provide antenatal care. Antenatal clinics are held once a week in all the health facilities and managed by a matron.

**Study population:** all consenting pregnant women at 20 weeks to 36 weeks of gestation who attended the antenatal clinic at least once in the selected PHCs from March to April 2015 were enrolled in the study.

**Study design and sampling:** a health facility-based cross-sectional study was conducted. The sample size was calculated using formula for single proportions, based on an estimate 15% as the proportion of pregnant women who took 2+ doses of SP during an ANC visit [9], standard normal deviate set at 1.96 (for 95% confidence level), 0.84 (for 80% power) and, precision of 0.05 and adjusted for non-response and missing data of 10% to give a total of 444 respondents.

$$N=\frac{{\left(Z_{\text{α}}+Z_{\text{β}}\right)}^{2}\text{pq}}{d^{2}}$$

**Figure 1:**
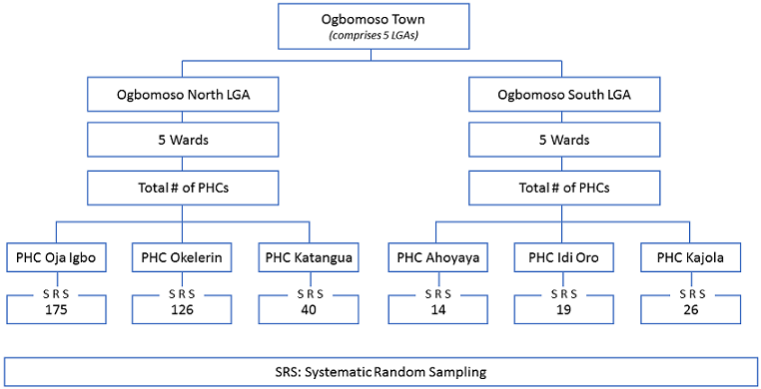
Sampling procedure for selection of pregnant women

…[14] N= (2.80)^2^ x 0.15 x 0.85/(0.05)^2^ = 400. Respondents were selected using a multi-staged sampling technique. In the first stage, two LGAs (Ogbomoso North and Ogbomoso South) were randomly selected using five LGAs in Ogbomoso town as sampling frame. In each selected LGA, five wards were randomly selected. From each of the selected wards, the list of PHCs was obtained from the respective LGA offices. Three PHCs offering antenatal care services were selected from the list of PHCs in the five wards by balloting, making a total of six PHCs. The average monthly antenatal clinics attendees in the selected PHCs were obtained from the health administration. The number of pregnant women to be interviewed from the selected PHCs was proportionately allocated based on the number of attendees. Systematic random sampling technique was used to select eligible respondents from each selected facility until the respective sample size was achieved (Figure 1).

### Data collection

**Quantitative:** trained research assistants administered questionnaire to obtain information on socio-demographic information, obstetric history, knowledge of IPTp-SP, attitude towards IPTp-SP use and utilisation of IPTp-SP. The dependent variable was utilisation of IPTp-SP while age of respondents, educational level, occupation, income, parity, marital status, knowledge on IPTp-SP, attitude towards IPTp-SP were independent variables.

**Qualitative:** the principal investigator and trained research assistants conducted six focus group discussion (FGD) sessions in the local language “Yoruba” using an FGD guide. The FGD guide focused on ANC booking period, concept of IPTp-SP, benefits and side effects of IPTp-SP medicines and availability and usage of IPTp-SP medicines. Each FGD session was conducted among eight purposively-selected pregnant women attending ANC in each of the six selected PHCs, 48 in all. Each audio-taped FGD session lasted for 45 to 60 minutes. Additionally, a note-taker captured information about the setting and non-verbal cues among discussants. Six key informant interviews (KII) were conducted with the matrons-in-charge of the antenatal clinics of the PHCs using a KII guide.

**Operational definitions:** in this study, the appropriate IPTp-SP utilisation was defined as the uptake of at least two doses of IPTp-SP between second trimester and early part of third trimester, while inappropriate IPTp-SP utilisation was the uptake of less than two doses of SP between second trimester and early part of third trimester. Directly observed treatment (DOT) was the direct observation of a pregnant woman by qualified health staff as she swallows SP at the antenatal clinic. Early ANC booking was booking from 1^st^ to 13^th^ week of pregnancy while late ANC booking was anytime from 14^th^ week of pregnancy. Minimum wage is an earning of N18,000 ($64) obtained on a monthly basis.

**Data analysis:** we entered and cleaned data using Statistical Package for Social Sciences (SPSS) (IBM Incorporation) version 16 software. Data were summarized using frequencies, means, and proportions. Odds ratio at 95% confidence interval (CI) was used to compare the strength of association for categorical variables like age group, occupation, educational status, respondents' knowledge and attitude to the uptake of IPTp-SP. Multivariate analysis was conducted to determine independent predictors of IPTp-SP use. The significant factors on bivariate analysis (occupation, number of ANC visits, knowledge on IPTp and attitude towards IPTp), were included in the logistic regression model. A 6-point question on knowledge on IPTp-SP was analysed as follows. A correct response to each question was scored one point, while an incorrect response was scored zero. Respondents who scored below 4 points were regarded as having poor knowledge of IPTp-SP while those who scored up to or above 4 points were regarded as having good knowledge of IPTp-SP. Attitude towards IPTp-SP use in pregnancy was scored on a 3-point Likert scale of agree, indifferent and disagree. Eleven questions about the attitude towards IPTp-SP were described with a mean attitude score of 26.3 ± 3.6. Respondents who scored below the mean were regarded as having a negative attitude towards IPTp-SP while those who scored up to or above the mean were regarded as having a positive attitude towards IPTp-SP. For the FGD reports, audio files were transcribed phonetically in the original language (Yoruba) and then translated into English. Sections of text were double-checked for accuracy of translation by other members of the field team. Transcripts were then analysed using the thematic approach. KII reports were also analyzed using the thematic approach.

## Results

**Figure 2:**
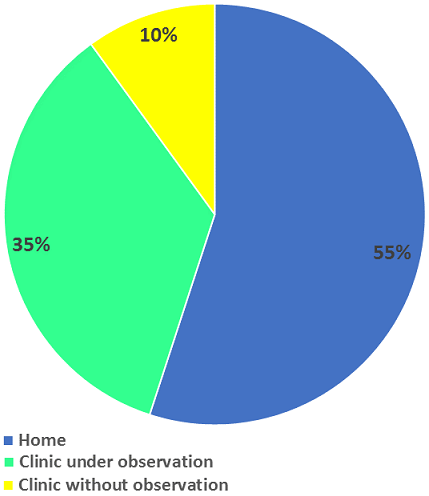
Adherence to DOT scheme

Overall, 400 respondents were included in the analysis. A total number of 430 questionnaires were administered during the study. Four hundred questionnaires were returned properly filled and four hundred questionnaires were analyzed, giving a response rate of 93%. A total of 244 (61.0%) respondents were in the 20-29 years' age group. A total of 381 (95.2%) were married, and 196 (49.0%) had secondary school education. One hundred and forty (35.0%) were skilled employees (artisans), and 281 (70.2%) had a monthly income of less than minimum wage. Among the study respondents, 269 (67.2%) were above 26 weeks at the time of the interview. Overall 211 respondents (52.8%) started attending ANC during their second trimester (Table 1). Most of the respondents of the focus group discussion said ANC booking should be from the second trimester (Table 2). *"Pregnant women start coming to the clinic early in pregnancy by 3 months so as to receive good care"* (FGD Oja-Igbo). Two hundred and ninety-four (73.5%) respondents had 2-4 clinic visits (Table 1). Overall, 320 (80.0%) respondents used IPTp-SP drugs at some point in their current pregnancy, while 80 (20.0%) did not. Of the 320 pregnant women who had taken at least one dose, 168 (52.5%) took only a dose of SP, while152 (47.5%) took two doses. Of the 320 pregnant women who had taken at least one dose; 63 (19.7%) took the first dose during the first trimester, 221 (69.1%) respondents took the first dose of SP during the second trimester, and 36 took the first dose (11.3%) during the third trimester (Table 3). In all, 112 (35.0%) of 320 respondents took their drugs under the direct observation of the health care worker at the health facility (DOT) (Figure 2). Regarding what DOT scheme meant, all the six health facility heads said it is Directly Observed Therapy, and it meant the administration of SP to an eligible pregnant woman in the presence of a healthcare worker at the clinic during the antenatal visit. However, the practice of DOT scheme from the FGD report was said not to be adhered to at the health facilities because SP was mostly out of stock in the health facilities (Table 2).*"The medicines are not usually available at the clinic. The last time I came to clinic I was not given the medicine that prevents against malaria, it was written out for me to go and buy and then use at home"* (PHC Oja-Igbo). Also, drug prescriptions given to pregnant women by healthcare workers were purchased from pharmacy stores and used at their homes.

**Table 1:** General characteristics and obstetric information of respondents attending antenatal clinics in primary health centers in Ogbomoso, Oyo State- 2015 (N=400)

Characteristics	Frequency (N)	Percentage (%)
**Age group (in years)**		
< 20	24	6.0
20-29	244	61.0
30-39	127	31.8
40-49	5	1.2
**Marital status**		
Single	17	4.3
Married	381	95.2
Divorced	2	0.5
**Educational status**		
No formal education	23	5.8
Primary education	36	9.0
Secondary education	196	49.0
Tertiary education	145	36.2
**Occupation**		
Unemployed	68	17.0
Student	43	10.8
Unskilled	88	22.0
Skilled	140	35.0
Professional	61	15.2
**Income (in naira)**		
<18,000	281	70.2
18,000 and above	119	29.8
**Gestational age (in weeks)**		
20-26	131	32.8
≥ 27	269	67.2
**Parity**		
0	132	33.0
1	111	27.8
≥ 2	157	39.2
**Gravidity**		
1	132	33.0
2-4	244	61.0
≥ 5	24	6.0
**Booking period**		
1^st^ trimester	105	26.2
2^nd^ trimester	211	52.8
3^rd^ trimester	84	21.0
**ANC visit**		
1	48	12.0
2-4	294	73.5
>4	58	14.5

**Table 2:** Participant responses on thematic areas during FGD in the selected health facilities

Themes	Responses	Oja-Igbo FGD 1	Kajola FGD 2	Katangua FGD 3	Idi-Oro FGD 4	Okelerin FGD 5	Ahoyaya FGD 6
ANC booking period	1^st^ trimester	-	++	+	-	-	-
	2^nd^ trimester	+++	+++	+++	+++	+++	+++
	3^rd^ trimester	-	-	-	+	-	-
Concept about IPTp	Prevention of malaria during pregnancy	+	++	++	+	+	++
Benefits of IPTp drugs	Couldn’t remember	++++	+++	+++	++	++++	+++
Side effects of IPTp drugs	No harm to the mother and baby	++++	++++	++++	++++	++++	++++
IPTp drugs availability and usage	SP not available	+++	+++	+++	+++	+++	+++
	SP mostly taken at home	+++	+++	+++	+++	+++	+++

Key: -- = None; + =Few (<25%); ++ = Some (50%); +++ =Majority (>75%); ++++All (100%)

**Table 3:** Utilization of intermittent preventive treatment among respondents attending antenatal clinics in primary health centers in Ogbomoso, Oyo State- 2015

Characteristics	Frequency (N)	Percentage (%)
**Use IPTp drugs (N=400)**		
Yes	320	80.0
No	80	20.0
**Number of SP dose used (N=320)**		
1	168	52.5
2	152	47.5
**Trimester 1^st^ dose SP (N=320)**		
1^st^	63	19.7
2^nd^	221	69.0
3^rd^	36	11.3

There was an association between IPTp-SP utilization and being employed, having more than two ANC visits, good knowledge on IPTp-SP and positive attitude towards IPTp-SP. IPTp-SP utilisation was associated with being employed (odds ratio [OR]= 1.97; 95% confidence interval [CI] 1.22 - 3.19), more than two ANC visits (OR= 17.08; 95% CI 4.08 - 71.47) and positive attitude to IPTp-SP (OR= 1.54; 95% CI 1.01 - 2.33) (Table 4). Also, good knowledge on IPTp-SP was associated with IPTp-SP utilisation (OR= 3.99; 95% CI 2.31 - 6.88). FGD report respondents' good knowledge on IPTp-SP as *"A form of treatment given to pregnant women to prevent the occurrence of malaria fever"* (PHC Kajola). Also *"Fansidar, as we were being told during our clinic visit is the recommended medicine for the prevention of malaria during pregnancy"* (PHC Katangua). KII report showed that the facility heads were knowledgeable about IPTp-SP. All the six health facility heads interviewed knew IPTp using SP as a chemoprophylaxis given to pregnant women after 13 weeks of gestation until the time of delivery at a monthly interval for the prevention of malaria. Concerning the national policy on IPTp, two out of six facility heads gave an update on the policy. One (from PHC Idi-Oro) said the practice of directly observed therapy of IPTp in which a pregnant woman takes the SP in the presence of the healthcare worker at the clinic should be observed. The second facility head (PHC Ahoyaya) said at least two doses of SP was used before; presently SP is given after 13 weeks of gestation till delivery. After controlling for respondents age the determinants of IPTp-SP utilization were more than one antenatal (ANC) visit (adjusted odds ratio [aOR] = 5.6; 95% CI: 1.2-26.6) and knowledge on IPTp (aOR=9.25; 95% CI: 5.4-16.0). Attitude to IPTp (aOR=2.1; 95% CI: 1.5 - 2.9) and being employed (aOR=1.4; 95% CI: 1.1 - 1.7) (Table 5).

**Table 4:** Association of utilization of intermittent preventive treatment and socio-demographic characteristics of respondents attending antenatal clinics in primary health centers in Ogbomoso, Oyo State- 2015

Characteristics	SP doses based on gestational age			OR (95% CI)
	Appropriate N (%)	Inappropriate N (%)	Total N (%)	
**Occupation**				
Employed	122 (42.2)	167 (57.8)	289 (100.0)	1.97 (1.22-3.19)
Unemployed	30 (27.0)	81 (73.0)	111 (100.0)	
**Number of ANC visits**				
Two or more times	150 (42.6)	202 (57.4)	352 (100.0)	17.08 (4.08-71.47)
Once	2 (4.2)	46 (95.8)	48 (100.0)	
**Knowledge on IPTp**				
Good	133 (45.7)	158 (54.3)	291 (100.0)	3.99 (2.31-6.88)
Poor	19 (17.4)	90 (82.6)	109 (100.0)	
**Attitude towards IPTp**				
Positive	99 (42.1)	136 (57.9)	235 (100.0)	1.54 (1.01-2.33)
Negative	53 (32.1)	112 (67.9)	165 (100.0)	
**Gestational age at time of booking (in weeks)**				
≥ 14	111 (37.6)	184 (62.4)	295 (100.0)	0.94 (0.60-1.49)
1-13	41 (39.0)	64 (61.0)	105 (100.0)	

**Table 5:** Respondents’ determinants of appropriate utilization of intermittent preventive treatment attending antenatal clinics in primary health centers in Ogbomoso, Oyo State -2015

Characteristics	Adjusted Odds Ratio (AOR)	95% CI	
		Lower	Upper
**ANC visits**			
Once (Ref)	1.00	-	-
Two times or more	5.64	1.19	26.63
**Knowledge on IPTp**			
Poor (Ref)	1.00	-	-
Good	9.25	5.36	15.97
**Attitude towards IPTp**			
Negative (Ref)	1.00	-	-
Positive	2.11	1.52	2.93
**Occupation**			
Unemployed (Ref)	1.00	-	-
Employed	1.37	1.08	1.73

## Discussion

We found that early booking for ANC, more than two visits to ANC, good knowledge and positive attitude towards IPTp and being employed were factors associated with IPTp-SP utilisation. Respondents' good knowledge on IPTp was further corroborated by FGD findings of SP being the drug of choice with no harmful effect on the pregnant woman and the baby yet unborn. Positive attitude towards IPTp use might be because of the good level of IPTp awareness, which was mostly through the health care provider. We also found that utilisation of IPTp-SP among study respondents was low. Four out of ten respondents used at least two doses of SP. The proportion of those who used at least two doses of SP in this study was higher than the 15.7% reported in the 2013 Nigeria Demographic and Health Survey [9]. However, it is unlike findings conducted in South West Nigeria where 78.0% received at least two doses of SP during pregnancy [15]. The low utilisation of IPT was corroborated by FGD findings which revealed that SP was mostly out of stock in most of the health facilities. Late booking for ANC among respondents in this study could also have contributed to poor utilisation of IPT. Findings from this study were similar to a study conducted in Senegal, [16] which reported that timing of IPTp was directly linked to the onset of ANC visit. On the same note, Van Ejik *et al.* [17] found that delayed attendance at ANC contributed to non-completion of IPTp-SP doses. We also found that there was non-adherence by health care workers to the practice of DOT scheme. The non-adherence to the practice of DOT scheme could also have contributed to poor IPTp-SP utilisation. Pregnant women from the FGD session said they mostly use their drugs at home; this was however in contrast to FGD report of pregnant women in Tabora village, Tanzania [8]. The observation that pregnant women occasionally throw away their SP tablets after leaving the ANC clinics underscores the need for measures to improve the implementation of DOT approach.

The association between socio-demographic characteristics of respondents and utilisation of intermittent preventive treatment showed that there was a statistically significant difference between some ANC visits, occupation, knowledge on IPTp and attitude towards IPTp with the utilisation of intermittent preventive treatment. The significant difference could be because repeated antenatal care visits increases the opportunity to use the recommended SP doses. Also, employed mothers may have been more able to meet the financial obligations of antenatal registration and attendance which in turn enhanced their IPTp-SP utilisation as compared to their unemployed counterparts. Unexpectedly, our study showed about one-fifth of the interviewed respondents received IPTp-SP in the first trimester. This practice is not in accordance with the national guidelines and calls for further investigation. Our findings are subject to limitations. First, the interviews and discussions were conducted at the heath facilities which might have resulted in information bias due to fear of disclosed information reaching the health care providers. This was however minimized by interviewing the pregnant women in a private area where information was collected confidentially. Second, there was the possibility of recall bias with regards to ANC visits and utilisation of IPTp-SP. This was however minimized by obtaining additional ANC information from health facility case files of respondents. The strength of the study was the use of qualitative data collection focus group discussion (FGD) allowed for more sensitive information to be collected.

## Conclusion

The findings of this study show that more than two visits to ANC, being employed, having good knowledge and positive attitude towards IPTp were determinants of IPTp-SP utilisation. Also, there was good knowledge and positive attitude towards IPT despite poor utilisation. Health workers need to be re-trained and adequately supervised to adhere to DOT scheme so as to improve IPTp-SP utilisation. Also, there should be continuous sensitization of pregnant women on the importance of intermittent preventive treatment during each pregnancy by healthcare workers.

### What is known about this topic

- Studies have shown low utilisation of intermittent preventive treatment during pregnancy across the geo-political zones of Nigeria with recent findings from the 2015 National Malaria Indicator Survey;
- The factors responsible for low utilisation of intermittent preventive treatment during pregnancy focuses on either the demand (client related) or supply (health system related) side, but not a mixed method approach in addressing low utilisation of intermittent preventive treatment during pregnancy.

### What this study adds

- This study adds that good knowledge and positive attitude towards IPTp, early booking for ANC, more than two visits to ANC and being employed were factors associated with IPTp-SP utilization;
- Government policy should enhance uninterrupted supply of SP in health facilities;
- There is a need for healthcare workers to adhere to directly observed treatment to improve IPTp-SP utilisation.

## Competing interests

The authors declare no competing interests.

## References

[1] World Health Organization (2016). World Malaria Report.

[2] Desai M, ter Kuile FO, Nosten F, McGready R, Asamoa K, Brabin B (2007). Epidemiology and burden of malaria in pregnancy. Lancet Infect Dis.

[3] Steketee RW, Nahlen BL, Parise ME, Menendez C (2001). The burden of malaria in pregnancy in malaria-endemic areas. Am J Trop Med Hyg.

[4] Ekejindu I, Udigwe G, Chijoke I (2006). Malaria and anemia in pregnancy in Enugu, southeast Nigeria. Afri J Med Sci.

[5] World Health Organization (2004). A strategic framework for malaria prevention and control during pregnancy in the Africa region.

[6] World Health Organization (2014). WHO policy brief for the implementation of intermittent preventive treatment of malaria in pregnancy using Sulfadoxine-Pyrimethamine (IPTp-SP).

[7] (2014). National guidelines and strategies for malaria prevention and control during pregnancy.

[8] Mubyazi G, Bloch P, Kamugisha M, Kitua A, Ijumba J (2005). Intermittent preventive treatment of malaria during pregnancy: a qualitative study of knowledge, attitudes and practices of district health managers, antenatal care staff and pregnant women in Korogwe district, Northern Eastern Tanzania. Malar J.

[9] National Population Commission (Nigeria) and ICF International (2014). Nigeria Demographic and Health Survey 2013.

[10] National Malaria Elimination Program (NMEP), National Population Commission (NPopC), National Bureau of Statistics (NBS) and ICF International (2016). Nigeria malaria indicator survey 2015: key indicators.

[11] Ameen S, Ajayi J (2013). Studies on influence of seasonality on clinical conditions of small ruminants in Ogbomoso areas of Oyo State. Int J Appl Agric Apic Res.

[12] Facts sheet on Ogbomoso (2016). World Heritage Encyclopedia.

[13] Department of Research and Statistics, Ministry of Economic Planning and Budget Ibadan, Oyo State (2014).

[14] Bamgboye E (2013). Lecture notes on research methodology in the health and medical sciences.

[15] Aduloju OP (2013). Effect of intermittent preventive treatment of malaria on the outcome of pregnancy among women attending antenatal clinic of a new Nigerian teaching hospital, Ado-Ekiti. Niger Med J.

[16] Olliaro P, Delenne H, Cisse M, Badiane M, Olliaro A, Vaillant M (2008). Implementation of intermittent preventive treatment in pregnancy with sulphadoxine/pyrimethamine (IPTp-SP) at a district health center in rural Senegal. Malar J.

[17] van Eijk AM, Ayisi JG, ter Kuile FO, Slutsker L, Otieno JA, Misore AO (2004). Implementation of intermittent preventive treatment with sulphadoxine-pyrimethamine for control of malaria in pregnancy in Kisumu, Western Kenya. Trop Med Int Health.

